# Antibiotics/coccidiostat exposure induces gut-brain axis remodeling for Akt/mTOR activation and BDNF-mediated neuroprotection in APEC-infected turkeys

**DOI:** 10.1016/j.psj.2024.104636

**Published:** 2024-12-04

**Authors:** Przemysław Sołek, Anna Stępniowska, Oliwia Koszła, Jan Jankowski, Katarzyna Ognik

**Affiliations:** a Department of Biochemistry and Toxicology, University of Life Sciences, Akademicka 13, 20-950 Lublin, Poland; b Department of Biopharmacy, Medical University of Lublin, Chodźki 4a, 20-093 Lublin, Poland; c Department of Poultry Science and Apiculture, University of Warmia and Mazury in Olsztyn, Oczapowskiego 5, 10-719 Olsztyn, Poland

**Keywords:** Antibiotics, Coccidiostats, APEC, Gut-brain axis, Neuroprotection

## Abstract

The poultry industry relies extensively on antibiotics and coccidiostats as essential tools for disease management and productivity enhancement. However, increasing concerns about antimicrobial resistance (AMR) and the toxicological safety of these substances have prompted a deeper examination of their broader impacts on animal and human health. This study investigates the toxicological effects of antibiotics and coccidiostats on the gut-brain axis and microbiota in turkeys, with a particular focus on molecular mechanisms that may influence neurochemical and inflammatory responses. Our findings reveal that enrofloxacin exposure leads to the upregulation of BDNF, suggesting a neuroprotective effect, while monensin treatment significantly increased eEF2 kinase expression, indicative enhanced neuronal activity. In turkeys infected with Avian Pathogenic *Escherichia coli* (APEC), early administration of doxycycline and monensin significantly upregulated the mTOR/BDNF and Akt/mTOR pathways, along with elevated histamine levels, underscoring their role in inflammatory responses modulation. However, treatments administered at 50 days post-hatch did not significantly alter protein levels, though both enrofloxacin and monensin increased serotonin and dopamine levels, suggesting potential neurotoxicological impacts on mood and cognitive functions. These results highlight the complex interactions between antibiotic use, gut microbiota alterations, and neurochemical pathways, with toxicological implications for environmental pollution and public health. This research provides critical insights into the potential toxic effects of prolonged antibiotic and coccidiostat exposure in poultry production, emphasizing the need for responsible use to mitigate risks to ecosystems and human health.

## Graphical abstract

**Figure:**
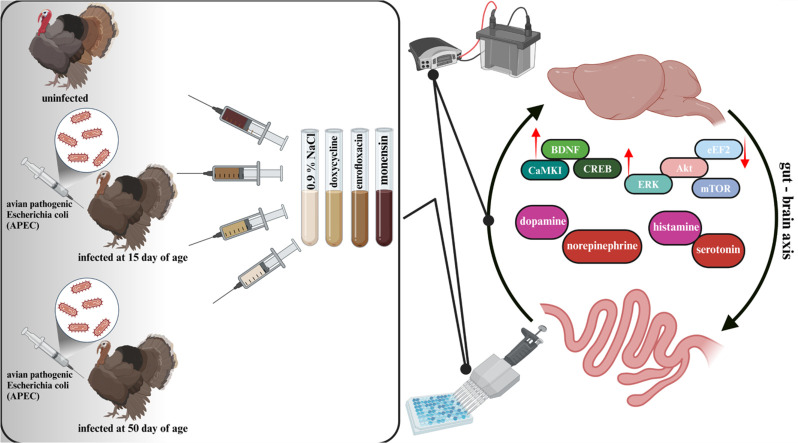

## Introduction

Antibiotics and coccidiostats have long been integral components of poultry farming practices aimed at health and productivity maintenance. Antibiotics are routinely administered to prevent and treat bacterial infections, while coccidiostats are used to control coccidiosis, a common intestinal disease. These interventions have historically been effective in reducing morbidity and mortality rates in poultry, thereby ensuring economic viability for producers (Abd El-Hack et al., 2022). Especially avian colibacillosis caused by pathogenic strains of Escherichia coli bacteria (APEC), stands out as a major threat to the poultry industry worldwide (Joseph et al., 2023).

However, concerns have arisen regarding the overuse and misuse of antibiotics in animal agriculture, leading to the emergence of antimicrobial resistance (AMR) among bacterial pathogens. More importantly, the indiscriminate use of antibiotics in poultry production systems has been implicated in the dissemination of resistant bacteria in both animals and humans, posing significant public health risks (Manyi-Loh et al., 2018). Similarly, the widespread use of coccidiostats in poultry farming has raised safety concerns, as these compounds can persist in the environment and potentially accumulate in the food chain, posing risks to human health (Martins et al., 2022).

The collection of diverse microorganisms have been found to have a crucial impact on multiple dimensions of animal well-being, going beyond its traditional functions in digesting food and absorbing nutrients (Hou et al., 2022). Moreover, between the gastrointestinal tract and the central nervous system (CNS) exists a bidirectional communication network that refers to the gut-brain axis, encompassing neural, hormonal, and immunological signaling pathways. This multifaceted interplay regulates various physiological processes, including immune function, and mood regulation. The gut microbiota affects gut-brain axis activity through neurotransmitters, metabolites, and signaling molecule production. Disruptions in gut-brain axis function have been implicated in the development of gastrointestinal and neurological disorders in animals, including poultry, where such imbalances can affect behavior, stress responses, and overall health (Beldowska et al., 2023; Calefi et al., 2016; Jadhav et al., 2022; Villageliu and Lyte, 2017). Disruptions in gut-brain axis function have been implicated in the pathogenesis of gastrointestinal disorders, neurodevelopmental conditions, and psychiatric disorders in humans and animals. In turn, hormonal signaling pathways imbalance can have profound effects on physiological homeostasis and may contribute to the development of metabolic disorders or behavioral disturbances.

Antibiotics can perturb the delicate balance of the gut microbiota, which in turn affects communication along the gut-brain axis as well as neurotransmitters secretion and has been associated with alterations in the mood and behavior of both poultry and humans (Hou, Wu, Chen, Wang, Zhang, Xiao, Zhu, Koya, Wei, Li and Chen, 2022). Also, coccidiostats can indirectly impact the gut microbiota and gut function, potentially influencing the gut-brain axis functions (Lu et al., 2021).

Altogether, within the realm of poultry production, the art of welfare management extends beyond mere maintenance. As our collective consciousness evolves, there is growing recognition that the health and welfare of farm animals are interconnected with broader societal concerns related to food safety, environmental sustainability, and ethical considerations (Macer, 2019).

The purpose of our study was to investigate the potential toxicological effects of early administration of antibiotics (enrofloxacin or doxycycline) and feeding turkeys a diet containing the coccidiostat (monensin) on the gut-brain axis and the levels of selected neurotransmitters, with a focus on the potential neurotoxic implications for mood regulation. Specifically, the study aimed to determine whether these interventions affect physiological and neurochemical responses in turkeys infected with APEC during either the early or later rearing periods. By examining how these commonly used substances in poultry farming influence both the neurological and gastrointestinal systems, we aim to uncover potential neurotoxic risks and provide insights into the broader toxicological consequences of antibiotic and coccidiostat use. This research contributes to our understanding of the complex toxicological interactions between the gastrointestinal system and the central nervous system in avian species, highlighting potential risks for animal health and environmental safety. Moreover, the current study is part of the same larger scientific project as Smagieł et al. (Smagieł et al., 2023), which investigated complementary aspects of turkey physiology under similar experimental conditions. Together, these studies offer a more comprehensive understanding of the systemic impacts of antibiotics and coccidiostats in poultry, emphasizing the need for cautious and responsible use to safeguard animal welfare, environmental health, and public safety.

## Materials and methods

Animal tests were carried out at the Animal Research Laboratory within the Department of Poultry Science and Apiculture at the University of Warmia and Mazury in Olsztyn, Poland. Approval for the study protocol was obtained from the Local Ethics Committee for Animal Experiments (resolution No. 47/2021), and the animals were subjected to care standards consistent with the principles outlined in EU Directive 2010/63/EU. The study was carried out in compliance with the ARRIVE guidelines. Every effort was made to minimize the suffering of the animals used in the experiment.

### Study design and treatment groups

The experimental design was previously described by Smagieł et al. (Smagieł, Ognik, Cholewińska, Stępniowska, Listos, Tykałowski, Mikulski, Koncicki and Jankowski, 2023). In detail, the study employed a randomized 4 × 3 factorial design with 4 groups. The first group included poultry treated with doxycycline (signed as D), enrofloxacin (signed as E), monensin (signed as M) and the control group without antibiotics. Poultry infected with early APEC were assigned to the next group, control (C-, 0.9% NaCl vehicle infection), doxycycline (D-, 0.9% NaCl vehicle infection), enrofloxacin (E-, 0.9% NaCl vehicle infection) and monensin (M-, 0.9% NaCl vehicle infection). The last groups involved experimental challenges of early infection with APEC at 15 days of age and late infection at 50 days of age, denoted as " APEC early infection" and " APEC late infection". Groups were signed as control (C+, early infection without antibiotics), doxycycline (D+, early infection), enrofloxacin (E+, early infection), monensin (M+, early infection) and control (C++, late infection without antibiotics), doxycycline (D++, late infection), enrofloxacin (E++, late infection) and monensin (M++, late infection).

### Experimental infection with APEC

In this experiment, we exclusively used female Hybrid Converter Novo turkeys across all groups. At both 15 and 50 days of age, eight turkeys were randomly chosen from each respective group (C, M, E, D) and subjected to infection with Avian Pathogenic Escherichia coli (APEC) using the methodology delineated by Mazur-Gonkowska et al. (Mazur-Gonkowska et al., 2004). Briefly, APEC strain (Escherichia coli O78:K80) with virulence factors: *cvi/cva+, vat-, tsh+, iucD+, papC+, irp2+, iss+, astA+*; provided by veterinary laboratory RB VAC (Zielona Góra, Poland). This strain was cultured under standard laboratory conditions to prepare it for the infection procedure. In detail, the APEC strain was cultured on Luria-Bertani (LB) agar plates (Thermo Fisher Scientific, USA) for 24 hours at 37°C to obtain individual colonies. Subsequently, a single colony was inoculated into LB broth (Oxoid) and incubated at 37°C with shaking at 200 rpm for 24 hours to reach the exponential growth phase. The bacterial culture was then diluted with sterile 0.9% NaCl solution to achieve the required concentration of about 4×10^5 colony-forming units (CFU) per turkey, which was administered via injection into the left abdominal air sac at a dosage of 4×10^5 colony-forming units (CFU) per turkey in 0.3 ml of 0.9% NaCl solution. Uninfected birds received 0.9% sterile NaCl solution in the abdominal air sac through the same route. Both infected and uninfected birds were housed in group pens located in separate sections of the facility and managed by distinct personnel to mitigate the risk of cross-contamination.

Infected and uninfected turkeys were housed in group pens located in distinct, segregated sections of the facility to prevent cross-contamination. Each pen accommodated 55 birds, contributing to a total of 385 birds per experimental group. Pens were uniformly lined with wood shavings, measured 10 m² in area, and maintained an initial stocking density of 5.5 birds/m². Environmental parameters, including temperature, humidity, and lighting, were controlled via automated systems and adjusted in accordance with the birds' age, following the management guidelines outlined by Hybrid Turkeys (Turkeys., 2020). Although infected and uninfected groups were housed separately, all birds were exposed to identical environmental conditions and husbandry practices, with ad libitum access to feed and water. These measures ensured uniformity in experimental conditions while rigorously maintaining biosecurity protocols. Throughout the study, the uninfected turkeys were rigorously monitored for potential signs of infection via systematic clinical observations. At no point were any clinical symptoms indicative of infection observed in turkeys, confirming their uninfected status under the conditions of the experiment. Additionally, mortality rates were recorded daily, and the detailed data are previously presented in Table 6 (Smagieł et al., 2024).

### Sample collection

Samples from all treatment subgroups were collected by birds euthanasia via cervical dislocation, following established guidelines for experimental animals. Hippocampal brain fragments and serum samples were obtained from eight individuals within each group. In detail, hippocampal brain tissue was collected immediately post-euthanasia. The hippocampus was precisely dissected using sterile instruments and immediately placed on dry ice to ensure rapid cooling and preservation of molecular and structural integrity. The tissue was subsequently stored at -80°C until further analysis to prevent postmortem degradation. In turn, blood samples were collected using sterile syringes, transferred into serum separator tubes, and allowed to clot at room temperature for 30 minutes. After centrifugation at 2000 g for 15 minutes at 4°C, the serum was separated, transferred into sterile tubes, and stored at -80°C.

### Protein quantification by enzyme-linked immunosorbent assay (ELISA)

Serum Plasma concentrations of dopamine (#QY-E80169), serotonin (#QY-E80171), noradrenaline (#QY-E80172) and histamine (#QY-E80170) were quantified in 7-21-56 days old turkeys using diagnostic kits from Qayee-Bio (Qayee Biotechnology, China) in accordance with manufacturer protocol. The absorbance readings were obtained at 450 nm wavelength using a microplate reader (Tecan, Switzerland). The concentration of the samples were interpolated from the standard curve.

### Protein expression quantification by western blot (WB) analysis

Hippocampal tissue was homogenized using bead mill technology to ensure efficient tissue disruption and preparation for Western blot analyses. Each sample was placed in 2 mL Bead Mill Tubes containing 200 mg of ceramic beads (1.0–1.2 mm in dimension) and 2% SDS supplemented with 1 mM PMSF freshly prepared. Homogenization was conducted using the Bead Mill Max Homogeniser (VWR International LLC, Radnor, USA) with the soft tissue program. The parameters were set at a speed of 5 m/s for 4 cycles, each lasting 45 seconds. To prevent overheating and potential degradation of biomolecules, samples were cooled on ice between cycles.

Total protein extracts from hippocampus tissue were prepared by automated homogenization (Bead Mill MAX) in 2% SDS buffer supplemented with 1 mM PMSF freshly prepared and 1,2mm zirconia beads. The homogenates were centrifuged and quantified using a protein determination (BCA) kit following Sołek et al. (Solek et al., 2021). Then, 20 µg of total protein extracts were resolved via SDS-PAGE electrophoresis, transferred onto methanol-activated PVDF membranes, blocked at room temperature (RT) in 1% BSA for an hour, followed by overnight incubation at 4°C with one of the primary antibodies targeting specific proteins, including anti-β-actin (1:1000, Cell Signaling, USA, #4967, RRID: AB_330288), anti-BDNF (1:1000, Affinity, UK, #DF6387, RRID: AB_2838350), anti-mTOR (1:1000, Cell Signaling, USA, #2972, RRID: AB_330978), anti-Akt (1:1000, Cell Signaling, USA, #4691, RRID: AB_915783), anti-eEF2 (1:1000, Cell Signaling, USA, #2332, RRID: AB_10693546), anti-ERK (1:1000, R&D System, USA, #MAB1940, RRID: AB_2140120), anti-CaMKI (1:5000, Abcam, USA, #ab68234, RRID: AB_1140889) and anti-CREB (1:1000, ThermoFisher, USA, #701120, RRID: AB_2532397) obtained from Thermo Scientific or CellSignaling. The protein of interest was probed with the secondary HRP-conjugated antibody, anti-mouse (#A9044) or anti-rabbit (#A0545) obtained from Sigma. Finally, the complexes were visualized using the ECL western blotting westar supernova substrate from Cyanagen, following the provided guidelines on the Azure 400 imaging system.

### Statistical analysis

The results are depicted as the mean ± SD. Data analysis employed one-way ANOVA followed by Dunnett's multiple comparison test, with significance set at p < 0.05.

## Results

### Antibiotics and coccidiostat affect specific mood-related biomarkers and molecular pathways

**Fig. 1:**
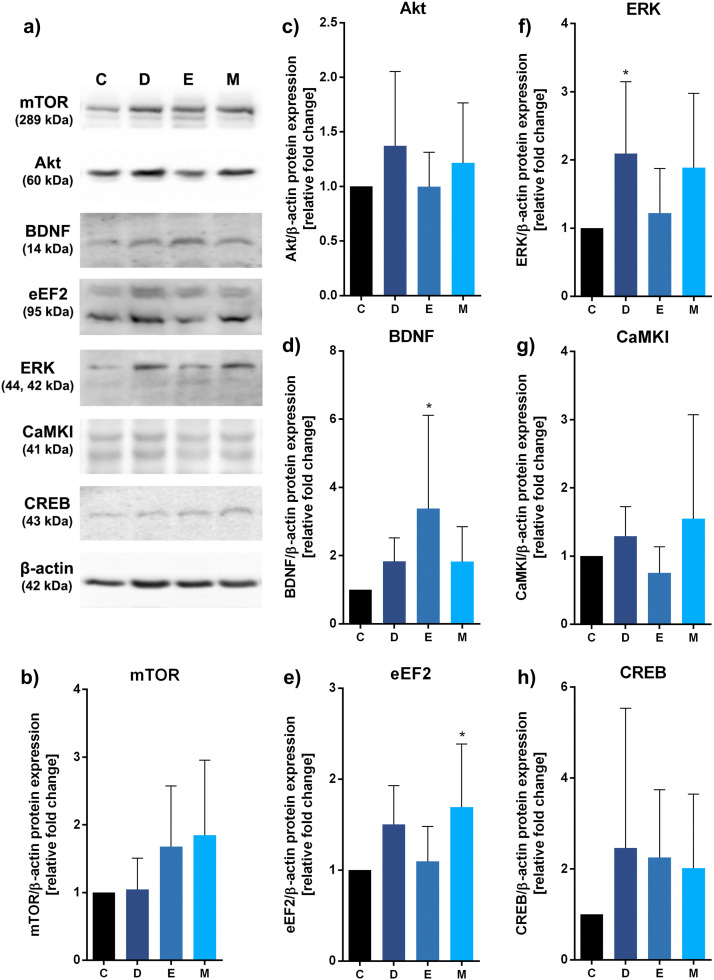
Impact of antibiotics and coccidiostat on molecular pathways related to the microbiota-gut-brain axis in turkeys treated with control (C, no antibiotic, uninfected), doxycycline (D, uninfected), enrofloxacin (E, uninfected), or monensin (M, uninfected). Selected protein levels were assessed: mTOR (B), Akt (C), BDNF (D), eEF2 (E), ERK (F), CaMKI (G), or CREB (H) and representative western blots are provided (A). Bars indicate mean and standard deviation (SD), n = 8, * p < 0.05.

**Fig. 2:**
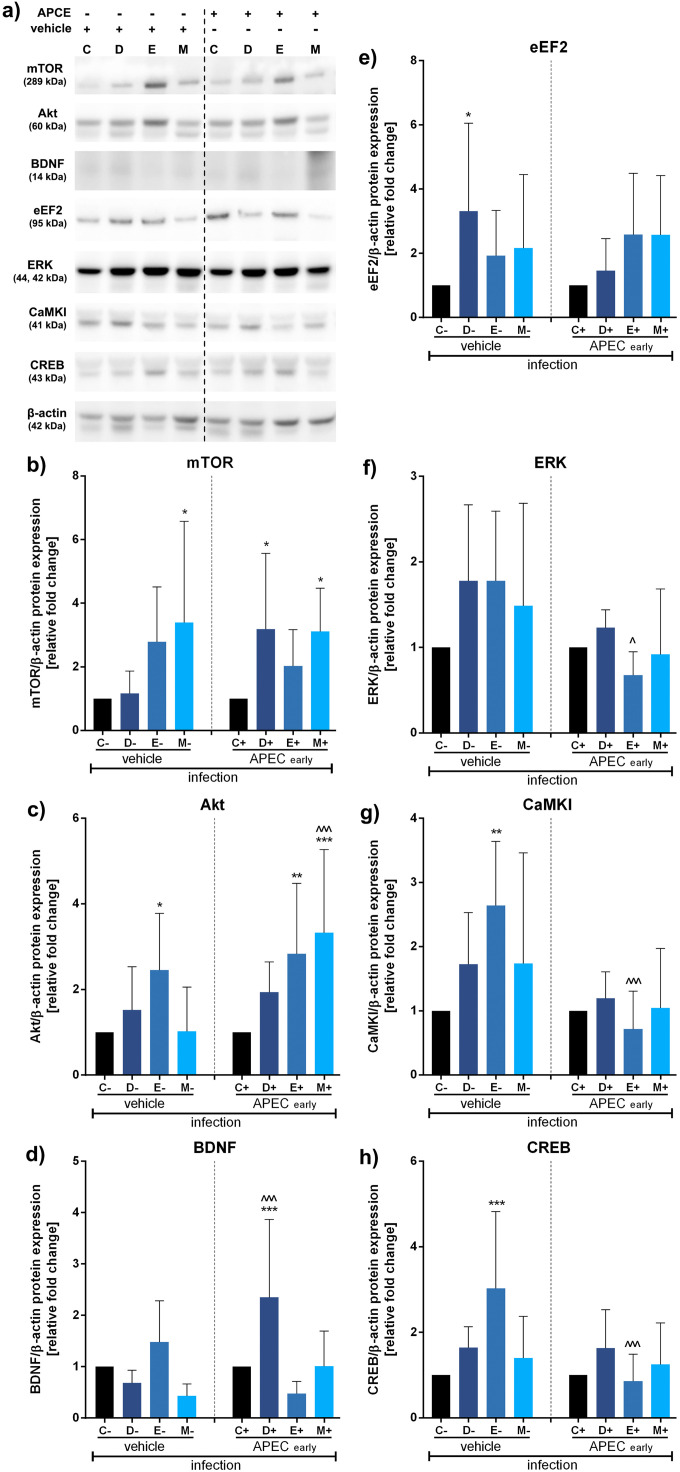
Impact of antibiotics and coccidiostat on molecular pathways related to the microbiota-gut-brain axis in turkeys uninfected (control group), infected at 15 days (early) or vehicle infected (0,9% NaCl) with APCE and treated with control (C, no antibiotic, uninfected), doxycycline (D, uninfected), enrofloxacin (E, uninfected), or monensin (M, uninfected). Selected protein levels were assessed: mTOR (B), Akt (C), BDNF (D), eEF2 (E), ERK (F), CaMKI (G), or CREB (H) and representative western blots are provided (A). Bars indicate mean and standard deviation (SD), n = 8, *^/^^ p < 0.05, ** p < 0.01, ***^/^^^^ p < 0.001.

**Fig. 3:**
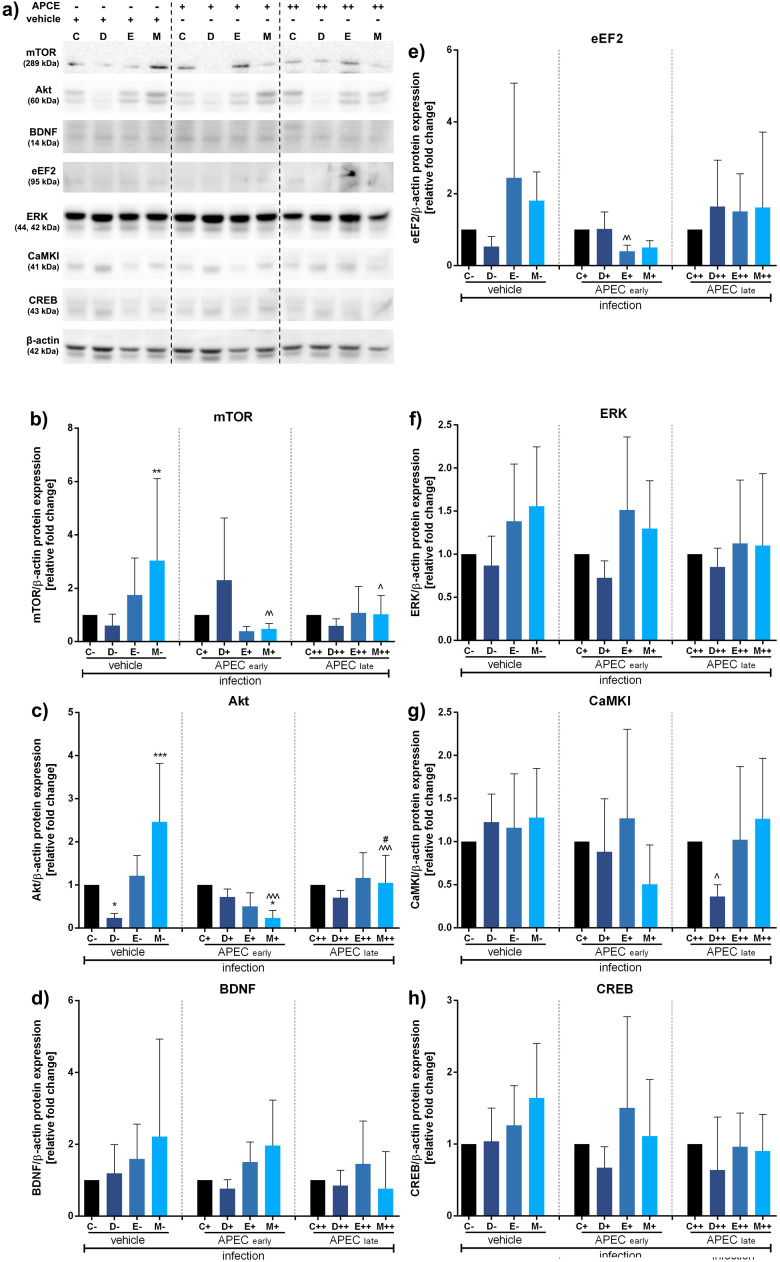
Impact of antibiotics and coccidiostat on molecular pathways related to the microbiota-gut-brain axis in turkeys uninfected (control group), infected at 15 (early) or 50 days (late) as well as vehicle infected (0,9% NaCl) with APCE and treated with control (C, no antibiotic, uninfected), doxycycline (D, uninfected), enrofloxacin (E, uninfected), or monensin (M, uninfected). Selected protein levels were assessed: mTOR (B), Akt (C), BDNF (D), eEF2 (E), ERK (F), CaMKI (G), or CREB (H) and representative western blots are provided (A). Bars indicate mean and standard deviation (SD), n = 8, *^/^^^/#^ p < 0.05, **^/^^^ p < 0.01, ***^/^^^^ p < 0.001.

In the first step of this study, we assessed the impact of antibiotics (enrofloxacin and doxycycline) or coccidiostat (monensin) on molecular pathways related to the microbiota-gut-brain axis (Figs. 1–3a). Our findings indicate that diets containing coccidiostat, with or without supplementation of doxycycline or enrofloxacin (uninfected), did not exert significant effects on mTOR (Fig. 1b), Akt (Fig. 1c), or CaMKI (Fig. 1g) kinases, nor CREB (Fig. 1h) transcription factor levels (ns, p>0.05, each). However, treatment with enrofloxacin (uninfected) resulted in BDNF upregulation (*, p<0.05), with no significant changes for doxycycline or monensin (Fig. 1d). The highest expression of eEF2 kinase was observed with monensin (*, p<0.05) (Fig. 1e), and for ERK with doxycycline (*, p<0.05) (Fig. 1f).

In the case of APEC infection at 15 days post-hatch, mTOR protein kinase was upregulated by monensin (vehicle and early infection, *, p<0.05, each) and doxycycline (early infection, *, p<0.05) (Fig. 2b). A similar pattern was observed for Akt kinase, with upregulation noted in the vehicle group for enrofloxacin (*, p<0.05) and in the early infected group for enrofloxacin (**, p<0.01) and monensin (***, p<0.001). Moreover, differences were observed between vehicle and APEC treatment groups for monensin (^^^, p<0.001) (Fig. 2c). BDNF transcription factor was upregulated only in the early infected, doxycycline-treated group (***, p<0.001) and in comparison between vehicle and APEC groups (^^^, p<0.001) (Fig. 2d). Changes in eEF2 and ERK kinases expression were also observed only in specific experimental sets, such as for doxycycline (eEF2, vehicle, *, p<0.05) and enrofloxacin (ERK, early infected, ^, p<0.05) compared to vehicle (Fig. 2, Fig. 2). Additionally, in the vehicle group, exposure to enrofloxacin resulted in CaMKI (**, p<0.01) and CREB (***, p<0.001) upregulation compared to control. In contrast, in the early APEC-infected group treated with enrofloxacin, significant CaMKI and CREB downregulation was observed compared to the vehicle group (^^^, p<0.001, both proteins) (Fig. 3, Fig. 3).

In the case of APCE infection at 50 days post-hatch, the observed changes were less pronounced. None of the treatments had a significant effect on BDNF (Fig. 3d), ERK (Fig. 3f) or CREB (Fig. 3h). However, monensin caused the most cumulative changes. Specifically, mTOR synthesis was upregulated compared to control (**, p<0.01), while it was downregulated after early (^^, p<0.01) and late APCE administration (^, p<0.05) compared to vehicle (Fig. 3b). A similar trend was noted for Akt kinase, with increased expression for monensin (***, p<0.001) but decreased for doxycycline (*, p<0.05) or monensin in the early infected group (*, p<0,05) compared to control. Interestingly, an increase was observed between early and late-infected groups (#, p<0.01) (Fig. 3c). Finally, regulation of eEF2 expression was observed only in specific experimental conditions, such as for enrofloxacin in early infection compared to vehicle (^^, p<0.01) (Fig. 3e) and for CaMKI with doxycycline in late infection compared to vehicle (^, p<0.05) (Fig. 3g).

### Antibiotics and coccidiostats regulate neurotransmitter release through changes in gut microbiota balance

**Fig. 4:**
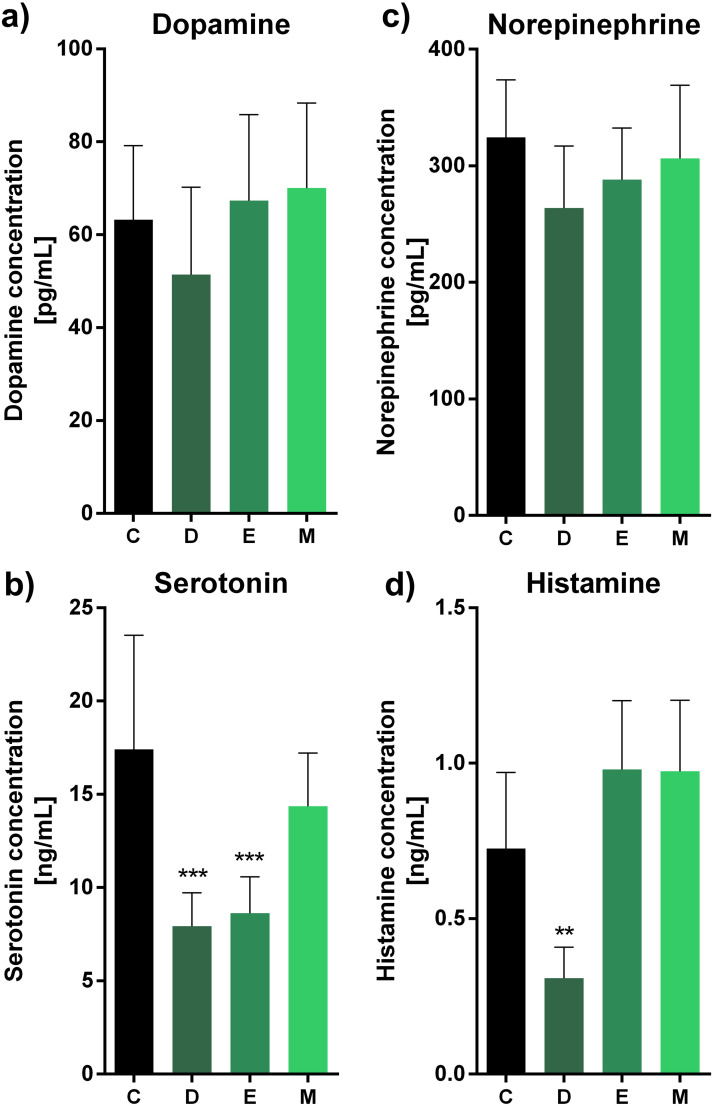
Impact of antibiotics and coccidiostat on selected neurotransmitters release in turkeys treated with control (C, no antibiotic, uninfected), doxycycline (D, uninfected), enrofloxacin (E, uninfected), or monensin (M, uninfected). Selected neurotransmitter levels were assessed: dopamine (A), serotonin (B), norepinephrine (C) or histamine (D). Bars indicate mean and standard deviation (SD), n = 8, ** p < 0.01, *** p < 0.001.

**Fig. 5:**
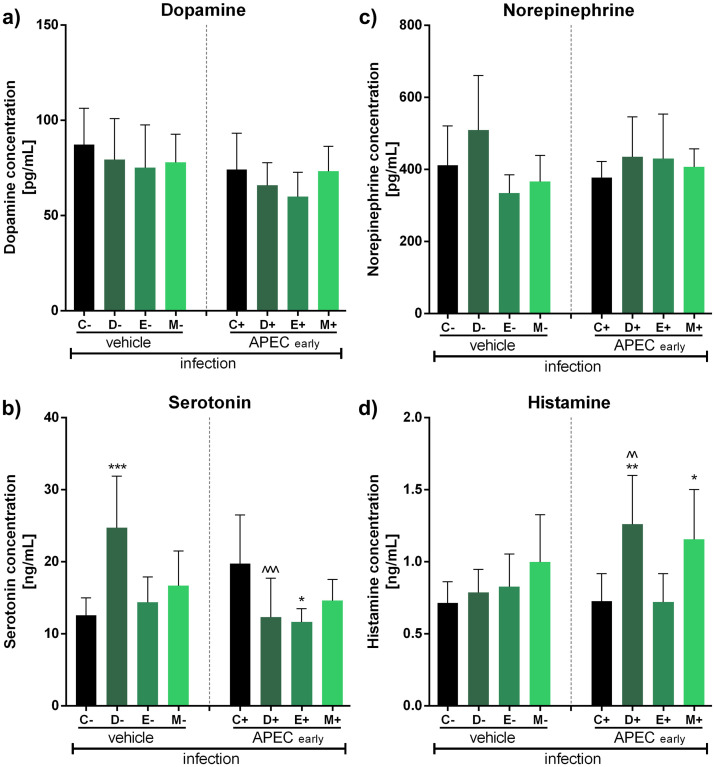
Impact of antibiotics and coccidiostat on selected neurotransmitters release in turkeys uninfected (control group), infected at 15 days (early) or vehicle infected (0,9% NaCl) with APCE and treated with control (C, no antibiotic, uninfected), doxycycline (D, uninfected), enrofloxacin (E, uninfected), or monensin (M, uninfected). Selected neurotransmitter levels were assessed: dopamine (A), serotonin (B), norepinephrine (C) or histamine (D). Bars indicate mean and standard deviation (SD), n = 8, * p < 0.05, **^/^^^ p < 0.01, ***^/^^^^ p < 0.001.

**Fig. 6:**
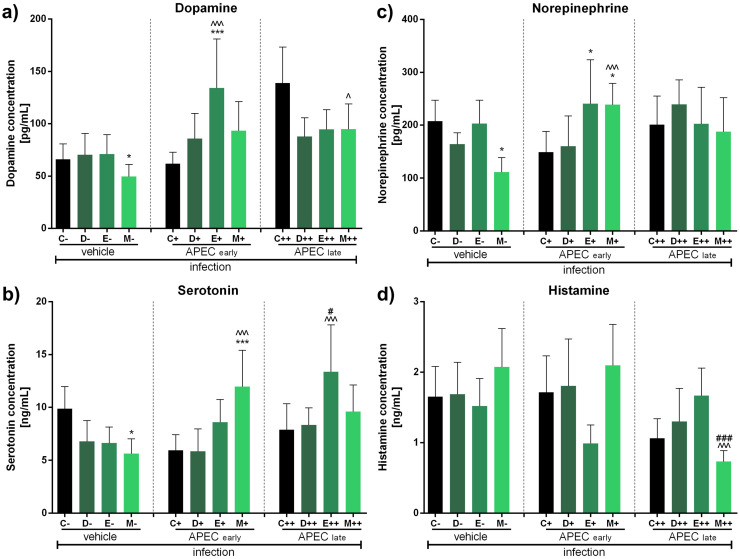
Impact of antibiotics and coccidiostat on selected neurotransmitters release in turkeys uninfected (control group), infected at 15 (early) or 50 days (late) as well as vehicle infected (0,9% NaCl) with APCE and treated with control (C, no antibiotic, uninfected), doxycycline (D, uninfected), enrofloxacin (E, uninfected), or monensin (M, uninfected). Selected neurotransmitter levels were assessed: dopamine (A), serotonin (B), norepinephrine (C) or histamine (D). Bars indicate mean and standard deviation (SD), n = 8, *^/^^^/#^ p < 0.05, ***^/^^^^^/###^ p < 0.001.

In the second step of this study, we examined the impact of antibiotics or coccidiostats on selected neurotransmitter release (Fig. 4, Fig. 5, Fig. 6). In uninfected groups, we observed a decrease in serotonin levels only for doxycycline and enrofloxacin (***, p<0.001, both) (Fig. 4b), and a decrease in histamine level only for doxycycline (**, p<0.01) (Fig. 4d). Interestingly, we did not observe significant changes in (Fig. 4a) or norepinephrine (Fig. 4c) levels in any of the experimental sets (ns, p>0.05, each).

Furthermore, in groups infected with APEC at 15 days post-hatch, no significant changes were observed in dopamine (Fig. 5a) or norepinephrine (Fig. 5c) levels. However, we observed a statistically significant increase in serotonin levels for doxycycline in the vehicle infection group (***, p<0.001) and conversely, a decrease for enrofloxacin in the early infection group (*, p<0.05) compared to control. Additionally, we observed a decrease in serotonin release following doxycycline treatment between the vehicle and early infection groups (^^^, p<0.001) (Fig. 5b). Moreover, an increase in histamine levels was observed. Specifically, in the control group for doxycycline (*, p<0.05) and monensin (**, p<0.01). Similarly, significant histamine upregulation was observed in the early infection group for these substances (**, p<0.01; *, p<0.05, respectively). Changes between the vehicle and early infection groups were only observed for doxycycline (^^, p<0.01) (Fig. 5d).

Finally, in groups infected with APEC at 50 days post-hatch, similar observations were made for dopamine, norepinephrine, and serotonin levels with monensin in the vehicle infection group (decrease, *, p<0.05 each). In contrast, an increase was observed for enrofloxacin in the early infection group for dopamine (***, p<0.001) and norepinephrine (*, p<0.05). Moreover, for monensin-treated groups, an increase was observed in the early infection group for norepinephrine (*, p<0.05) and serotonin (***, p<0.001) related to control. Increases in dopamine levels were noted between the vehicle and early or late infection groups for enrofloxacin (early, ^^^, p<0.001) and monensin (late, ^, p<0.05) (Fig. 6a), for norepinephrine with monensin-treated (early, ^^^, p<0.001) (Fig. 6b), and for serotonin with monensin-treated (early, ^^^, p<0.001) and enrofloxacin (late, ^^^, p<0.001) (Fig. 6c). Lastly, regarding histamine, no significant changes were observed compared to control. Only a decrease in monensin levels was observed in the APEC late infection group relative to early monensin (###, p<0.001) and relative to vehicle infection (^^^, p<0.001) (Fig. 6d).

## Discussion

The intricate relationship between the gastrointestinal tract and the central nervous system has garnered increasing attention in the field of animal science, particularly concerning its implications for animal health. Here we investigated the effects of antibiotics and coccidiostats administration to turkeys infected or not with Avian Pathogenic Escherichia coli (APEC) on molecular pathways associated with the microbiota-gut-brain axis and the release of neurotransmitters, which are indicative of mood and cognition alterations. Moreover, our study is situated within a complex landscape shaped by various factors influencing poultry productivity and the potential consequences for animal welfare.

Antibiotics or coccidiostats exert profound effects on gut bacteria composition and thus have a significant impact on the bidirectional communication system linking the gut microbiota with the central nervous system, influencing various aspects of neurological function and behavior. Data proved that antibiotics can disrupt this delicate balance by altering the diversity and abundance of gut microbiota, leading to dysbiosis and subsequent effects on brain function (Dahiya and Nigam, 2023; Ramirez et al., 2020). In turn, coccidiostats may also indirectly impact the gut microbiota through their antimicrobial properties (Künzel et al., 2019), thus potentially altering brain function. Moreover, dysbiosis has been associated with changes in neurotransmitter levels (Hayer et al., 2024), neuroinflammation (Solanki et al., 2023), and alterations in behavior in poultry (Fu and Cheng, 2024) including anxiety and depression-like symptoms.

In the first experimental part on uninfected birds, we observed increased BDNF, eEF2, and ERK expression in individual groups exposed to substances tested. Simultaneously, in peripheral blood, we noted a decrease in serotonin levels in doxycycline and enrofloxacin groups, as well as histamine levels in doxycycline group (antibiotics only). Also, we evaluated the effects of enrofloxacin, doxycycline or monensin in turkeys infected with APEC which is a prevalent risk in poultry production. Turkeys were exposed to the pathogen either at 15 or 50 days post-hatch, representing early and later stages of development, to simulate stressors encountered during different phases of rearing. Interestingly, in total, we observed the most changes 15 days compared to 50 days post-infection. In particular, these changes involved mTOR, BDNF, Akt, eEF2, CaMKI, and CREB-dependent signaling cascades in groups treated with monensin or enrofloxacin, and the fewest for doxycycline 15 days after infection. Additionally, increases in neurotransmitter levels, namely serotonin and histamine, were evident but only in doxycycline and monensin–treated groups. Lastly, assessment at 50 days post-hatch revealed less pronounced changes, with monensin inducing the most cumulative alterations in mTOR and Akt synthesis. In the case of neurotransmitters, decreases in dopamine, norepinephrine and serotonin levels were also noted for monensin in the vehicle group and remained unchanged 50 days after infection.

Changes in the composition of gut microorganisms can affect their ability to produce neurotransmitters and neuroactive chemicals acting CNS. Antibiotics can also influence the reduction in the activity or number of enterochromaffin cells (EC) or the sensitivity of neurotransmitter receptors at the CNS level, leading to decreased synthesis and release of neurotransmitters such as serotonin and histamine (Jadhav, Han, Fasina and Harrison, 2022). Finally, these effects can be mediated, at least in part, by microbial metabolites, such as short-chain fatty acids (SCFAs), which can modulate neuroinflammatory pathways and neurotransmitter synthesis (Lyte et al., 2024). SCFAs can modulate neuroinflammatory pathways by interacting with G protein-coupled receptors (GPCRs) such as GPR41, GPR43, and have been implicated in the regulation of neurotransmitter synthesis and release through the CNS (Mirzaei et al., 2021). Additionally, SCFAs proved to regulate the activity of enzymes involved in neurotransmitter synthesis, such as tyrosine hydroxylase and tryptophan hydroxylase, which are responsible for the production of dopamine and serotonin (Lyte, Eckenberger, Keane, Robinson, Bacon, Assumpcao, Donoghue, Liyanage, Daniels, Caputi and Lyte, 2024). This is not all, because SCFAs such as acetate, propionate, and butyrate, have the capacity to interact with a wide array of genes associated with the dopaminergic and serotonergic systems (Jadhav, Han, Fasina and Harrison, 2022), including dopamine receptors (D1, D2, D3, D4, and D5) (van de Wouw et al., 2018) and serotonin receptors (5-HT1A, 5-HT1B, 5-HT2A, 5-HT2C) (Buey et al., 2023), along with the glutamatergic system, including NMDA and AMPA receptors (NMDARs, AMPARs), glutamate transporters, and associated enzymes (Baj et al., 2019), as well as genes involved in managing the hypothalamic-pituitary-adrenal (HPA) axis, which plays a critical role in the stress response and mood regulation (Hamamah et al., 2022). Furthermore, antibiotics have been shown to affect the integrity of the intestinal barrier, leading to increased permeability and the translocation of bacterial products into the systemic circulation (Stolfi et al., 2022). Overall, it seems that SCFAs represent a crucial link between the gut microbiota and a number of brain disorders, serving as signaling molecules that communicate between the intestinal environment and CNS (Mansuy-Aubert and Ravussin, 2023).

BDNF neurotrophic factor and eEF2/ERK kinases interact with mentioned neurotransmitter systems to regulate various aspects of brain function, including mood, cognition, and stress responses. Especially, BDNF, eEF2 and ERK kinases are involved in synaptic plasticity thus high levels of BDNF may be reflected in the growth and survival of neurons and the formation of new synaptic connections (Yang et al., 2020; Yang et al., 2022). Additionally, ERK activation can lead to the regulation of genes expression associated with neuroplasticity (Fan et al., 2023), and eEF2 activation can influence the rate of protein translation, ultimately impacting neuron function, communication and mood (Monteggia et al., 2013). BDNF expression may also indicate a potential neuroprotective effect while alterations in eEF2 and ERK kinases may reflect an adaptive cellular response (Bathina and Das, 2015) to signals from the gut exposed to antibiotics or coccidiostats, or infection with APEC, aiming to maintain cellular homeostasis and function or influence neuroprocesses that are important for long-term memory and adaptation to stress. Thus, it seems that overlapping effects of synaptic activity and gut-brain axis activation by the microbiome may contribute to mood improvement (Beldowska, Barszcz and Dunislawska, 2023).

Moreover, APEC infection may induce stress responses and disrupt the production or metabolism of neurotransmitters (Kathayat et al., 2021), however, our findings suggest positive response of the studied birds suggests that the observed effects contribute to the absence of cognitive impairment through the activation of adaptive mechanisms to changing conditions. Additionally, APEC infection may trigger a robust immune response, leading to the release of pro-inflammatory cytokines and activation of immune cells but the mentioned kinases are part of cell signaling pathways that may control various processes, including proliferation and differentiation (Smagieł, Ognik, Cholewińska, Stępniowska, Listos, Tykałowski, Mikulski, Koncicki and Jankowski, 2023). This explanation, although partially reflected in the expression of mTOR in most experimental systems, is likely associated with cell proliferation control, protein synthesis, and energy metabolism regulation (Hao et al., 2021). It is worth noting that observed disruptions in mTOR expression following exposure to APEC, both in early and late stages, underscore the complex temporal dynamics of molecular responses to infection and treatment. These changes in mTOR expression may result from various adaptive or compensatory mechanisms that the organism activates in response to bacterial infection and/or the antibiotics or coccidiostats action (Afzal et al., 2022). This may include both pro-inflammatory and anti-inflammatory responses, depending on the context and timing of the intervention. At the same time, the precise mechanisms regulating mTOR expression in response to APEC infection remain to be investigated and may involve interactions with other signaling pathways and the influence of the microbiological gut environment. Furthermore, differential responses in dopamine, norepinephrine, and serotonin levels were noted between antibiotic and coccidiostat treatments, indicating distinct neurochemical profiles associated with each intervention. Notably, serotonin levels increased following doxycycline treatment in early APEC infection, suggesting a potential role in mitigating infection-induced behavioral changes. Taken together, our findings underscore the multifaceted impact of antibiotics and coccidiostats on neurotransmitter systems, with implications for mood regulation and cognitive function in infected birds. Importantly, the effects of antibiotics on the microbiota-gut-brain axis may have long-lasting consequences, particularly when exposure occurs during critical developmental periods. Early-life antibiotic use has been linked to alterations in brain development, cognitive function, and susceptibility to neuropsychiatric disorders later in life.

## Conclusion

In summary, our study elucidates the intricate interplay between antibiotics, coccidiostats, molecular pathways, and neurotransmitter regulation in turkeys infected with APEC, with particular emphasis on the toxicological effects of these substances. While antibiotics and coccidiostats demonstrate differential impacts on molecular pathways associated with the microbiota-gut-brain axis, they also modulate neurotransmitter release, potentially leading to neurotoxic outcomes affecting mood and cognitive function. Importantly, our findings revealed no immediate negative toxicological changes, particularly over the birds extended lifespan, likely due to the activation of adaptive mechanisms that mitigate toxic effects. These results underscore the necessity of evaluating both molecular and behavioral outcomes to assess the toxicological safety and therapeutic efficacy of antibiotics and coccidiostats in poultry production. Further mechanistic research is highly warranted to clarify the complex toxicological interactions within the microbiota-gut-brain axis and to inform targeted interventions aimed at minimizing toxic risks while optimizing poultry health and welfare.

## Authors contributions

PS: Conceptualization, Methodology, Formal analysis, Investigation, Resources, Data curation, Writing—original draft preparation, Writing—review and editing, Visualization,. AS: Methodology, Investigation. OK: Methodology, Investigation, Software, Formal analysis, Writing—review and editing. JJ: Conceptualization, Methodology, Supervision, Project administration. KO: Conceptualization, Investigation, Formal analysis, Resources, Writing—original draft preparation, Writing—review and editing, Supervision, Project administration, Funding acquisition.

All authors read and approved the final version of this manuscript.

## Declaration of competing interest

The authors declare the following financial interests/personal relationships which may be considered as potential competing interests:

Jan Jankowski reports financial support was provided by National Science Centre Poland. If there are other authors, they declare that they have no known competing financial interests or personal relationships that could have appeared to influence the work reported in this paper.

## Data Availability

The datasets used during the current study are available from the corresponding author on reasonable request.
